# Genome-wide analysis of *HSP70* family genes in cabbage (*Brassica oleracea* var. *capitata*) reveals their involvement in floral development

**DOI:** 10.1186/s12864-019-5757-3

**Published:** 2019-05-14

**Authors:** Henan Su, Miaomiao Xing, Xing Liu, Zhiyuan Fang, Limei Yang, Mu Zhuang, Yangyong Zhang, Yong Wang, Honghao Lv

**Affiliations:** Institute of Vegetables and Flowers, Chinese Academy of Agricultural Sciences, Key Laboratory of Biology and Genetic Improvement of Horticultural Crops, Ministry of Agriculture, Beijing, 100081 China

**Keywords:** Cabbage, Genome-wide, *HSP70* family genes, Expression profiling

## Abstract

**Background:**

Heat shock proteins have important functions in regulating plant growth and response to abiotic stress. *HSP70* family genes have been described in several plant species, but a comprehensive analysis of the *HSP70* family genes in cabbage has not been reported to date, especially their roles in floral development.

**Results:**

In this study, we identified 52 *BoHSP70* genes in cabbage. The gene structures, motifs, and chromosome locations of the *BoHSP70* genes were analyzed. The genes were divided into seven classes using a phylogenetic analysis. An expression analysis showed that the *BoHSP70* genes were highly expressed in actively growing tissues, including buds and calluses. In addition, six *BoHSP70* genes were highly expressed in the binuclear-pollen-stage buds of a male fertile line compared with its near isogenic sterile line. These results were further verified using qRT-PCR. Subcellular localization analysis of the bud-specific gene *BoHSP70–5* showed that it was localized in the cytoplasm.

**Conclusions:**

Our results help to elucidate the involvement of the *BoHSP70* family genes in cabbage floral development and establish the groundwork for future research on the functions of these genes.

**Electronic supplementary material:**

The online version of this article (10.1186/s12864-019-5757-3) contains supplementary material, which is available to authorized users.

## Background

HSP70, a type of heat shock protein (HSP), has long been recognized as one of the most conserved protein families, which can respond to external environmental stimuli and improve the ability of the organism to adjust to an adverse environment [1–3]. HSP70s function as molecular chaperones, presumably by protecting proteins against aggregation based on their ability to bind to hydrophobic amino acid residues or surfaces that are exposed by proteins in nonnative states. HSP70s are found in almost all organisms and are highly conserved [4–6]. Under normal conditions, the heat shock protein accounts for approximately 5% of the total cellular protein, but when the organism is subjected to environmental stress, especially high temperature conditions, it can account for up to 15% of the total cell protein [7]. HSPs can be divided into five families: HSP100, HSP90, HSP70, HSP60 and sHSP based on their molecular weights; among them, the HSP70 family is the most widely distributed HSP in vivo, which is not only the most studied but also the most conservative in terms of evolution [8]. The HSP70 family has drawn the greatest attention, because it is fundamental to plant developmental processes and functions during heat stress when the organism is subjected to external stress [9].

Many studies have shown that *HSP70* is closely related to plant abiotic stress [10], disease resistance [11], and growth and development [12]. When the plant suffers from high temperature, drought, high salt, low temperature, and heavy metals, HSP70s rapidly accumulate to maintain the stability of the protein and biological macromolecules to improve the resistance of the plant [7]. In addition, some studies found that the HSP protein has some relationship with plant embryogenesis. Cordewener et.al studied the embryonic development of *Brassica napus* revealing that heat shock at 32 °C for 8 h was associated with a few de novo synthetic 70-kDa HSPs: HSP68 and HSP70, the HSP70 family was upregulated by heat shock stimulation [13]. The relationship between heat shock treatment and embryogenesis was also studied in *Brassica napus*, and HSP70 and HSP90 located in the nucleus and cytoplasm were found to be rapidly induced [14].

There are relatively few studies on the roles of HSP70s in the development of organisms, HSP70s are also essential during normal growth. Duck et al. found that HSP70 transcripts were detected in mature anthers in tomato [15]. Sung et al. found that *HSP70–1*, *− 2*, and *− 3* were widely expressed in the roots, leaves, stems, flowers, and siliques, but *HSP70–4* was only expressed in roots and leaves, and *HSP70–5* was not detected in any tissues of *Arabidopsis* [16]. The cpHSP70s maintain chloroplast structure and function and orchestrate plant development [17, 18]. In addition, cpHSP70s and mtHSP70s act as part of a chaperone to help precursor proteins translocate to their individual destinations [19].

*HSP70* has been identified in many plants. *Arabidopsis* contains at least 17 genes encoding members of the HSP70 family proteins [16], while at least 26 members in rice [20], 12 members in spinach [21], and 61 putative *HSP70* members in soybean were found [22]. Cabbage (*Brassica. oleracea* var. *capitata*) is one of the most important vegetable crops in the world. As quite few studies have been conducted on cabbage *HSP70* family genes, we know little about their functions in growth and the response to environmental stress tolerance to heat shock. Thus, a genome-wide analysis of the *BoHSP70* genes will help to reveal the underlying complex molecular mechanisms. The publication of the genome data of *Brassica* enables the systematic analyses of *HSP70* evolution and function. In this study, the bioinformatics method was used to analyze genomic *HSP70* gene family members of cabbage, including the number of genes, chromosomal localization, phylogenetic relationships, structural features, functional predictions and expression analysis. These results lay the groundwork for the functional identification of the *HSP70* genes and its application in breeding more adaptable cabbage cultivars.

## Results

### Genome-wide identification of the *BoHSP70* family genes in cabbage

A total of 52 *BoHSP70* genes were identified and designated *BoHSP70–1*-*BoHSP70–52* using consecutive nomenclature. Detailed information about each *BoHSP70* gene is shown in Table 1. The *BoHSP70s* encoded proteins varied from 107 to 896 amino acids (aa) in length. Among these proteins, the BoHSP70–28 protein sequence was the shortest with 107 amino acids, and the BoHSP70–1 protein sequence was the longest with 896 amino acids. Forty of the *BoHSP70* genes were distributed on all nine chromosomes with chromosomes 1 and 3 harboring the most (nine, respectively) *BoHSP70* genes. The other 12 genes were located on different scaffolds and were not mapped to any chromosome. Within the 52 BoHSP70 proteins, 32 members shared the similar localization to cytosol, two to ER, ten to mitochondria, two to chloroplast, two to nucleus membrane, and four members were located in more than one compartment.

**Table 1:** *HSP70* family genes in the cabbage genome

No.	Gene ID	Name	Protein	Gene	Chr	Tandem	Localization predicted
			Length (aa)	Length (bp)			
1	Bol029014	BoHSP70–1	896	4901	C01	NO	Mit
2	Bol009554	BoHSP70–2	437	2912	C01	YES	Chl
3	Bol009553	BoHSP70–3	414	2895	C01	YES	Mit
4	Bol039526	BoHSP70–4	370	1762	C01	NO	Cyt
5	Bol018771	BoHSP70–5	377	1622	C01	NO	Cyt
6	Bol036512	BoHSP70–6	280	2355	C01	NO	Mit
7	Bol030974	BoHSP70–7	377	1377	C01	NO	Cyt
8	Bol007329	BoHSP70–8	271	2384	C01	NO	Mit
9	Bol023082	BoHSP70–9	354	1855	C01	NO	N
10	Bol024627	BoHSP70–10	377	1376	C02	NO	Cyt
11	Bol015484	BoHSP70–11	377	1668	C02	NO	N
12	Bol012377	BoHSP70–12	259	861	C02	NO	Cyt
13	Bol008775	BoHSP70–13	377	1397	C03	NO	Cyt
14	Bol025936	BoHSP70–14	651	3666	C03	NO	Cyt
15	Bol004630	BoHSP70–15	373	2724	C03	NO	ER
16	Bol005557	BoHSP70–16	588	2627	C03	NO	ER
17	Bol005539	BoHSP70–17	377	1739	C03	NO	Cyt
18	Bol022870	BoHSP70–18	377	1299	C03	NO	Cyt
19	Bol041394	BoHSP70–19	377	1433	C03	NO	Cyt
20	Bol032741	BoHSP70–20	613	1985	C03	NO	Mit
21	Bol035016	BoHSP70–21	466	3337	C03	NO	Cyt
22	Bol025569	BoHSP70–22	377	2531	C04	NO	Cyt
23	Bol044280	BoHSP70–23	259	1545	C04	NO	Mit
24	Bol014092	BoHSP70–24	563	1692	C04	NO	Mit
25	Bol025245	BoHSP70–25	377	1290	C04	NO	Cyt
26	Bol030773	BoHSP70–26	692	6667	C05	NO	Cyt
27	Bol010298	BoHSP70–27	365	2280	C05	NO	Cyt
28	Bol010299	BoHSP70–28	107	324	C05	NO	Cyt
29	Bol010323	BoHSP70–29	377	1688	C05	NO	Cyt
30	Bol005376	BoHSP70–30	377	1438	C06	NO	Cyt
31	Bol031604	BoHSP70–31	377	1413	C06	NO	Cyt
32	Bol042853	BoHSP70–32	360	2336	C07	NO	Cyt
33	Bol042165	BoHSP70–33	433	2957	C07	NO	Chl
34	Bol013215	BoHSP70–34	331	3699	C08	NO	Cyt
35	Bol025147	BoHSP70–35	377	1552	C08	NO	Cyt
36	Bol045633	BoHSP70–36	357	1568	C08	NO	Cyt
37	Bol006580	BoHSP70–37	395	2536	C08	NO	ER/Mit/N
38	Bol032207	BoHSP70–38	389	2732	C09	NO	Cyt
39	Bol017308	BoHSP70–39	586	4491	C09	NO	Cyt
40	Bol043724	BoHSP70–40	377	1394	C09	NO	Cyt
41	Bol041704	BoHSP70–41	377	1396	Scaffold000009_P1	NO	Cyt
42	Bol037535	BoHSP70–42	363	1795	Scaffold000024	NO	Cyt
43	Bol036345	BoHSP70–43	301	2723	Scaffold000029	NO	ER/N
44	Bol023729	BoHSP70–44	112	4378	Scaffold000098	NO	Mit/N
45	Bol011835	BoHSP70–45	215	648	Scaffold000206	NO	Mit
46	Bol011837	BoHSP70–46	308	927	Scaffold000206	NO	Mit
47	Bol003943	BoHSP70–47	169	1213	Scaffold000335	NO	Cyt
48	Bol003401	BoHSP70–48	360	1733	Scaffold000351	NO	Cyt
49	Bol003004	BoHSP70–49	377	1372	Scaffold000367	NO	Cyt
50	Bol002682	BoHSP70–50	367	2872	Scaffold000379	NO	Cyt
51	Bol002311	BoHSP70–51	143	931	Scaffold000394	NO	Chl/N
52	Bol001093	BoHSP70–52	377	1605	Scaffold000470	NO	Cyt

**Fig. 1:**
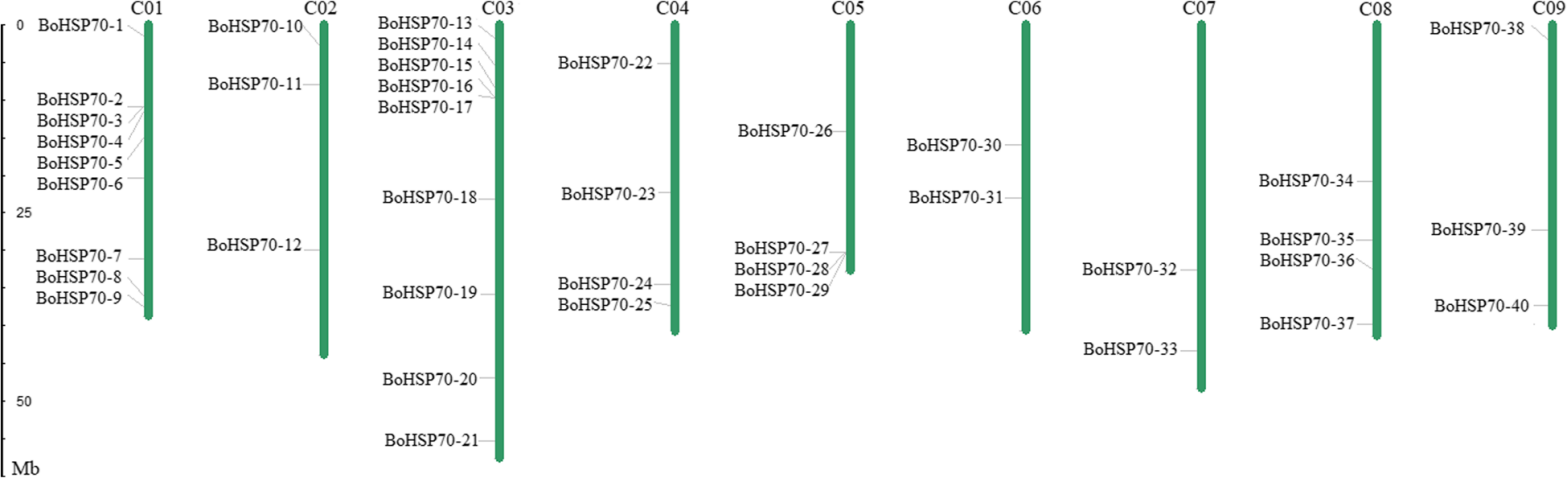
Distribution of the *BoHSP70* gene family members on the cabbage chromosomes. The bar located on the left side indicates the chromosome sizes in mega bases (Mb), and the scale represents the length of the chromosome

To show an overview distribution of the *BoHSP70* family members, a total of 40 genes were mapped onto the nine chromosomes in cabbage (Fig. 1). Most of these genes are anchored on nine chromosomes of cabbage, primarily distributed in the chromosomal middle and end regions. The numbers of *BoHSP70* genes on each chromosome are as follows: 9 on C01, 9 on C03, 4 on C04, 4 on C05, 4 on C08, 3 on C02, 3 on C09, 2 on C06, and 2 on C07. The remaining 12 genes, including *BoHSP70–41*-*BoHSP70–52*, were not distributed on the chromosome and were located on different scaffolds.

### Duplication and Ks analysis of the *BoHSP70* genes in cabbage

**Fig. 2:**
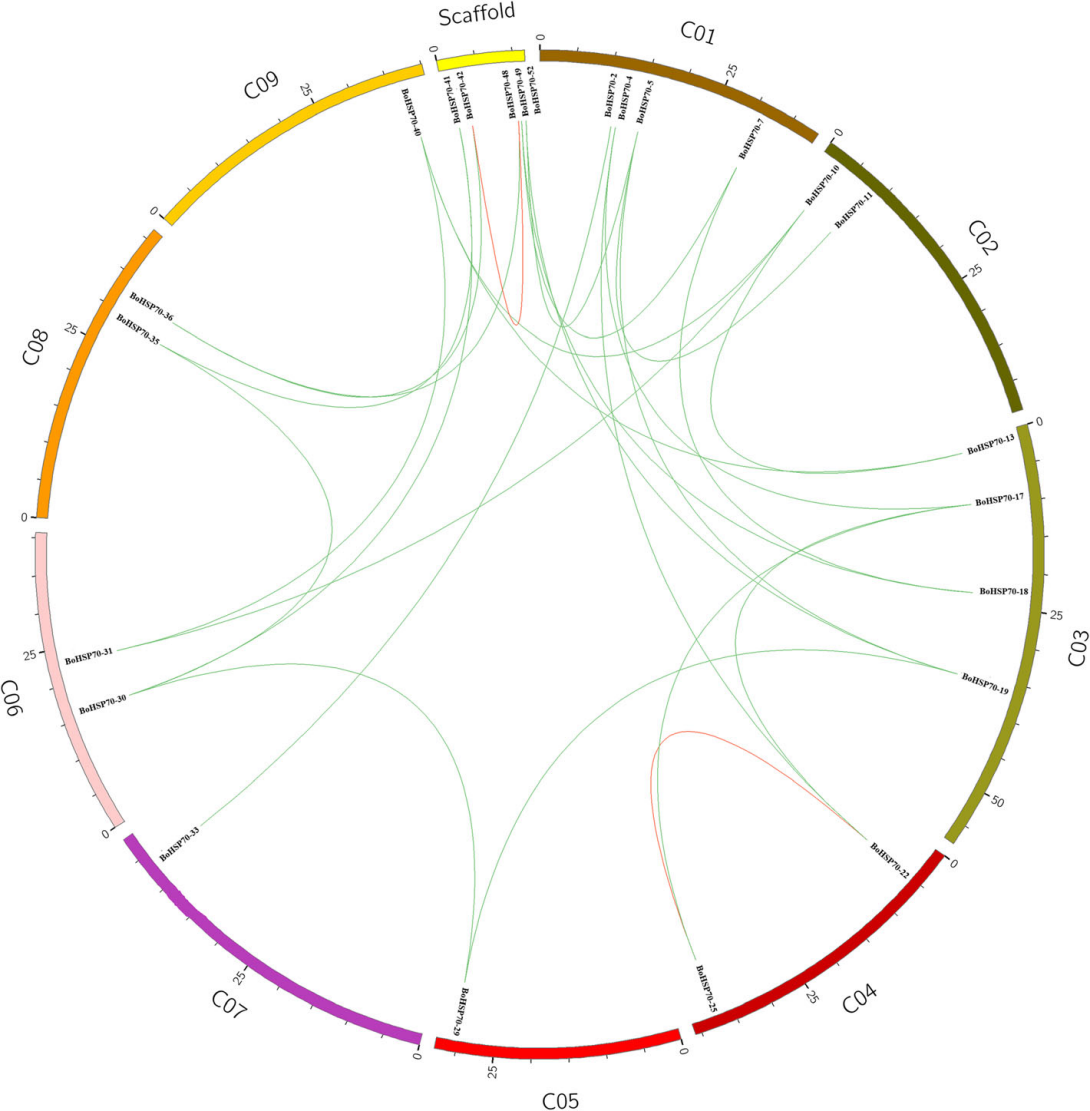
Duplication of the *BoHSP70* gene family members in cabbage

Gene duplication is one of the most important characteristics of plant genomic structure, which can occur by independent mechanisms resulting in segmental or tandem duplications. Due to the importance of gene duplications on the evolution of gene families in plants, we analyzed gene duplication of putative *BoHSP70* genes in the cabbage genome. We detected one tandem and 25 segmented duplicated gene couples among the 52 identified *BoHSP70* genes in cabbage (Fig. 2). A tandem duplicated gene couple was *BoHSP70–25/BoHSP70–22*, and segmental duplicated gene couples included *BoHSP70–4/BoHSP70–22*, *BoHSP70–13/BoHSP70–10*, *BoHSP70–25/BoHSP70–17*, *BoHSP70–52/BoHSP70–5*, *BoHSP70–52/BoHSP70–19*, *BoHSP70–13/BoHSP70–40*, *BoHSP70–5/BoHSP70–19*, *BoHSP70–30/BoHSP70–41*, *BoHSP70–35/BoHSP70–30*, *BoHSP70–31/BoHSP70–10*, *BoHSP70–35/BoHSP70–41*, *BoHSP70–10/BoHSP70–40*, *BoHSP70–17/BoHSP70–22*, *BoHSP70–4/BoHSP70–17*, *BoHSP70–18/BoHSP70–7*, *BoHSP70–49/BoHSP70–7*, *BoHSP70–49/BoHSP70–18*, *BoHSP70–31/BoHSP70–40*, *BoHSP70–29/BoHSP70–30*, *BoHSP70–29/BoHSP70–19*, *BoHSP70–11/BoHSP70–5*, *BoHSP70–36/BoHSP70–48*, *BoHSP70–36/BoHSP70–42*, *BoHSP70–33/BoHSP70–2* and *BoHSP70–48/BoHSP70–42*.

The value of Ka/Ks can be used as an indicator for the selection pressure of a gene during evolution. Our results indicated that all the values were less than 1, indicating that the *BoHSP70* genes primarily evolved under the influence of purifying selection (Table 2).

**Table 2:** Ks analysis of the *BoHSP70* genes in cabbage

Paralogous pairs	Ka	Ks	Ka/Ks	MYA
BoHSP70–25/BoHSP70–22	0.00468499	0.616754	0.0075962	44.31
BoHSP70–4/BoHSP70–22	0.0114715	0.311382	0.0368405	22.37
BoHSP70–13/BoHSP70–10	0.00460242	0.510366	0.00901789	36.66
BoHSP70–25/BoHSP70–17	0.00515522	0.600754	0.00858124	43.16
BoHSP70–52/BoHSP70–5	0.00379167	0.393786	0.00962874	28.29
BoHSP70–52/BoHSP70–19	0.00250496	0.418668	0.00598315	30.08
BoHSP70–13/BoHSP70–40	0.00473091	0.410763	0.0115174	29.51
BoHSP70–5/BoHSP70–19	0.0011933	0.393576	0.00303194	28.27
BoHSP70–30/BoHSP70–41	0.00507645	0.338896	0.0149794	24.35
BoHSP70–35/BoHSP70–30	0.00460708	0.356858	0.0129101	25.64
BoHSP70–31/BoHSP70–10	0.0288687	2.691	0.0107279	193.32
BoHSP70–35/BoHSP70–41	0.00239095	0.371426	0.00643723	26.68
BoHSP70–10/BoHSP70–40	0.00232753	0.373628	0.00622953	26.84
BoHSP70–17/BoHSP70–22	0.00530025	0.2989	0.0177325	21.47
BoHSP70–4/BoHSP70–17	0.00990488	0.13301	0.0744675	9.56
BoHSP70–18/BoHSP70–7	0.00133357	0.262495	0.00508038	18.86
BoHSP70–49/BoHSP70–7	0.00026549	0.265494	0.001	19.07
BoHSP70–49/BoHSP70–18	0.00117001	0.238548	0.0049047	17.14
BoHSP70–31/BoHSP70–40	0.0313242	2.7135	0.0115439	194.94
BoHSP70–29/BoHSP70–30	0.0287632	1.59633	0.0180184	114.68
BoHSP70–29/BoHSP70–19	0.0199692	1.70793	0.011692	122.7
BoHSP70–11/BoHSP70–5	0.00111277	1.0399	0.00107008	74.71
BoHSP70–36/BoHSP70–48	0.938473	1.1962	0.784544	85.93
BoHSP70–36/BoHSP70–42	0.928543	1.23367	0.752667	88.63
BoHSP70–33/BoHSP70–2	0.0425914	0.477849	0.0891316	34.33
BoHSP70–48/BoHSP70–42	0.0383699	0.327275	0.11724	23.51

### Phylogenetic relationship of the *BoHSP70* genes in cabbage

**Fig. 3:**
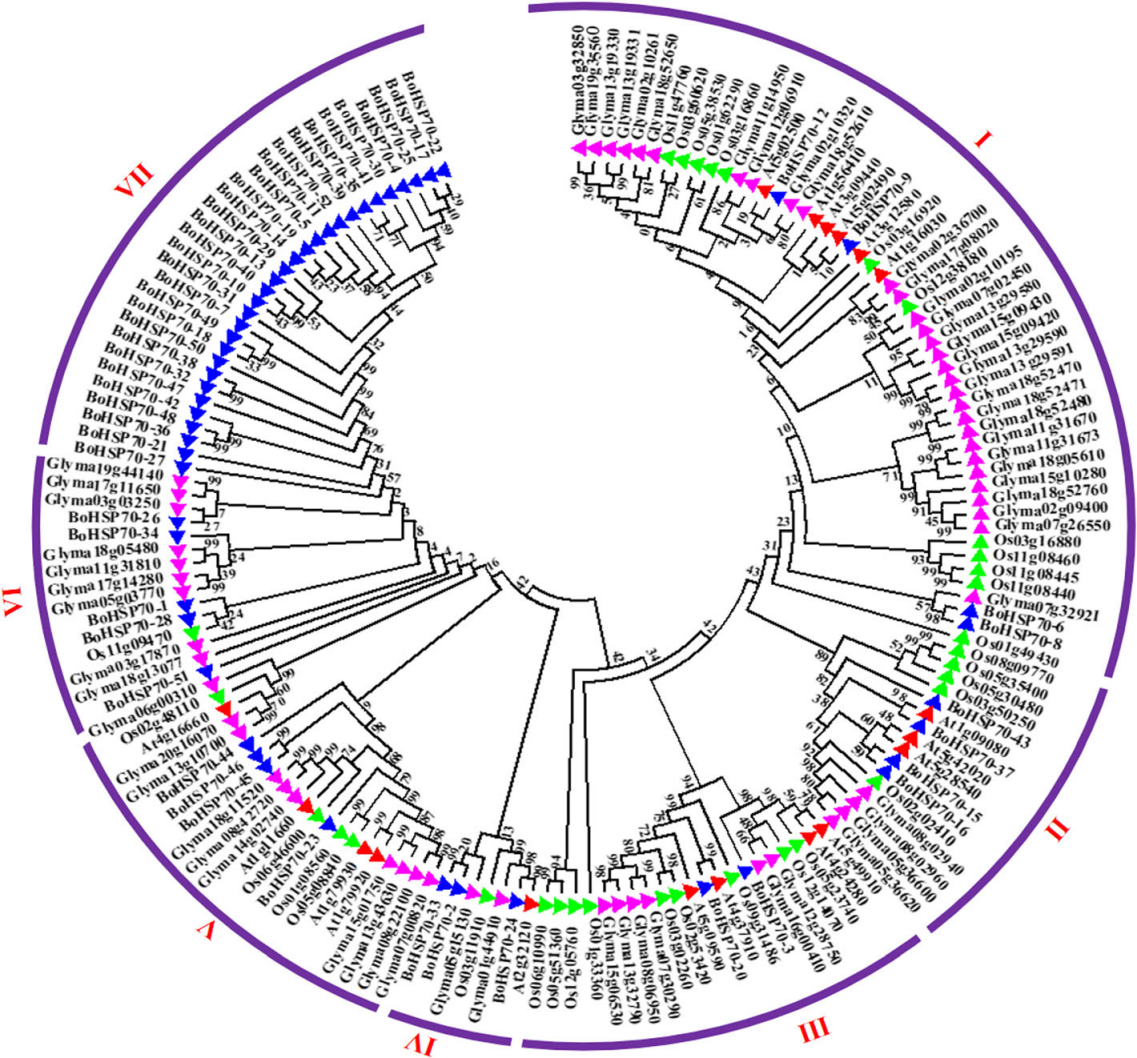
Phylogenetic tree of the HSP70 proteins from cabbage, Arabidopsis, rice, soybean. For the cabbage (prefixed by Bo) HSP70 proteins, gene names were used; the IDs of Arabidopsis (prefixed by At), rice (prefixed by Os), and soybean (prefixed by Glyma) HSP70 proteins were used

Based on the amino acid sequence of cabbage (52), soybean (61), Arabidopsis (18), and rice (32) HSP70 proteins, the BoHSP70 proteins phylogenetic tree of the *HSP70* family genes was constructed using software MEGA 5.0. According to previous studies, the *AtHSP70* gene family is divided into five sub-families, rice contained six sub-families, soybean contained eight sub-families. Therefore, based on their phylogenetic relationships, the combined cabbage, soybean, rice, and Arabidopsis phylogenetic trees can be divided into seven sub-families (class I-VII; Fig. 3). Among the seven clusters, class I was the largest, containing 52 members, and composed of four members from cabbage, 30 from soybean, 12 from rice, and six from Arabidopsis. Class II contained 16 members (four cabbage, four soybean, five rice, and three Arabidopsis members). The HSP70 members in Class III were two cabbage, six soybean, nine rice, and four in Arabidopsis. Class IV was a small sub-family, which only included three cabbage, one Arabidopsis, two soybean, and one rice member. Class V contained four cabbage, ten soybean, four rice, and four Arabidopsis members. Class VI contained five cabbage, one rice and nine soybean members. Class VII only contained 30 cabbage members, no soybean, rice, and Arabidopsis members. Excepted Class VII, all of the other sub-families contained rice, Arabidopsis, and soybean *HSP70* genes, those 30 cabbage members in Class VII are all located in the cytoplasm. In Class I-V, 18 members from Arabidopsis are mostly related to external stress, At5g49910 and At4g37910 act redundantly in the thermotolerance of germinating seeds, BoHSP70–12, 9, 6, 8, 43, 37, 15, 16, 3, 20, 24, 2, 33, 44, 45, and 46 may be related to stress, other family members in Class VI and VII may have other unknown functions.

### Structural analysis of the *BoHSP70* genes

**Fig. 4:**
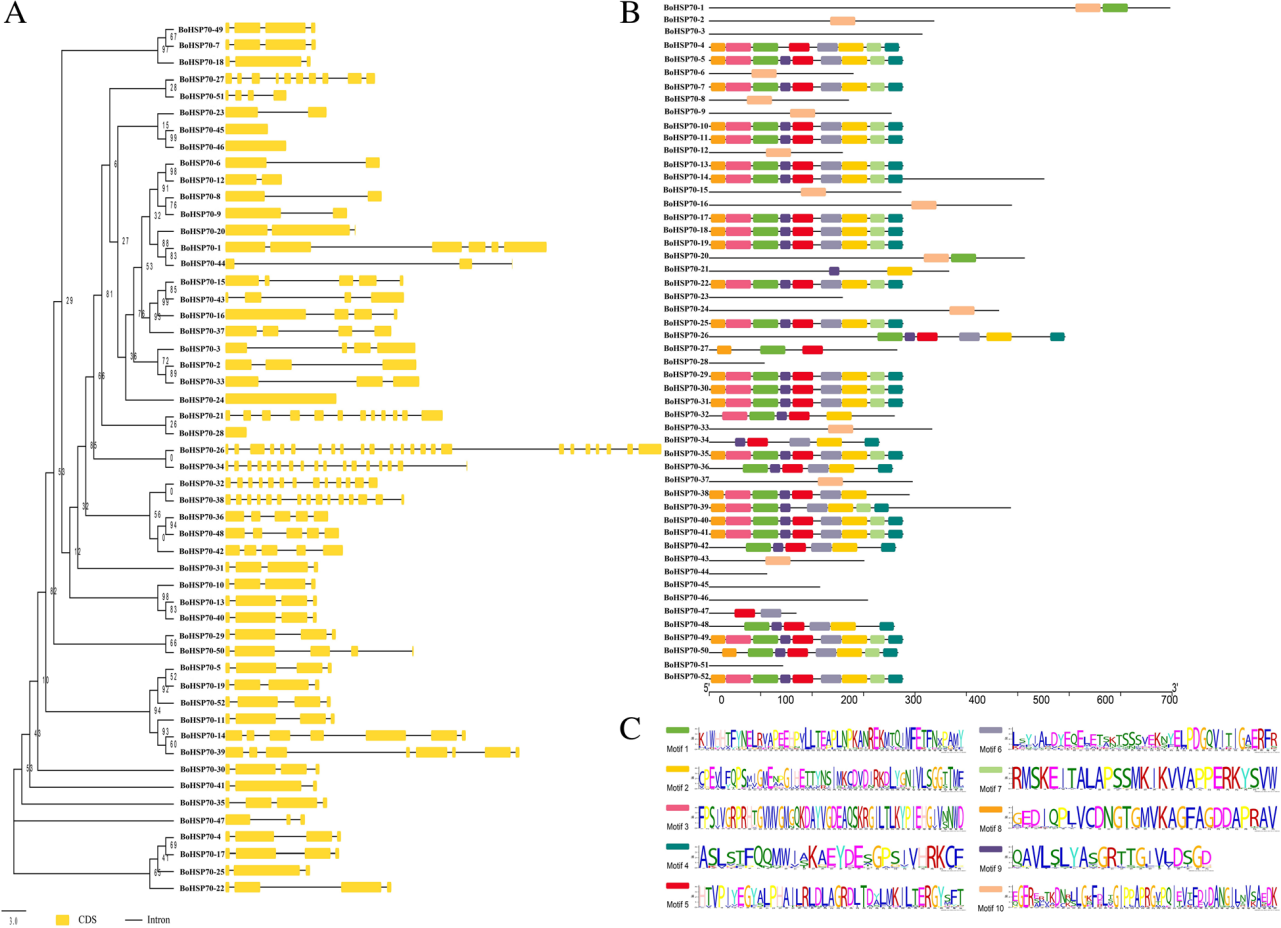
Gene structure and conserved motif analysis of the *BoHSP70* genes. **a** Gene structure of the *BoHSP70* genes in cabbage. **b** Motif compositions of *BoHSP70*. **c** Conserved motifs in *BoHSP70* were detected with MEME. Ten different motifs are represented by variously colored boxes

Based on the coding sequence of the *BoHSP70* genes, the structure of these genes was plotted using the online tool GSDS. Figure 4 provides a detailed illustration of the relative lengths of the introns and the conservation of the corresponding exon sequences within each *BoHSP70* gene. The number of introns in all these genes ranged from 0 to 23. Most of the *BoHSP70* genes contain three to eight introns, while *BoHSP70–26* has 23 introns, *BoHSP70–45* and *BoHSP70–46* lack introns. There are also large differences in the position and length of the introns.

To better understand the structural characteristics of the BoHSP70 proteins, ten consensus motifs were found in the BoHSP70 proteins using the MEME motif search tool, and the distribution of these conserved motifs in the BoHSP70 proteins was analyzed further. The results showed that most of these genes contained eight conserved motifs, which is missing up to 45 genes. Most of the closely related genes exhibit similar motif compositions, suggesting functional similarities in the *BoHSP70* family. The *BoHSP70–1*, *BoHSP70–2*, *BoHSP70–6*, *BoHSP70–8*, *BoHSP70–9*, *BoHSP70–12*, *BoHSP70–15*, *BoHSP70–16*, *BoHSP70–20*, *BoHSP70–21*, *BoHSP70–24*, *BoHSP70–33*, *BoHSP70–37*, *BoHSP70–43*, and *BoHSP70–47* genes had only one to two motifs. These results imply that the composition of the structural motifs varies among different members of the *BoHSP70* family genes but is similar within closely related genes.

### Expression patterns of the *BoHSP70* genes in various tissues and qRT-PCR validation

**Fig. 5:**
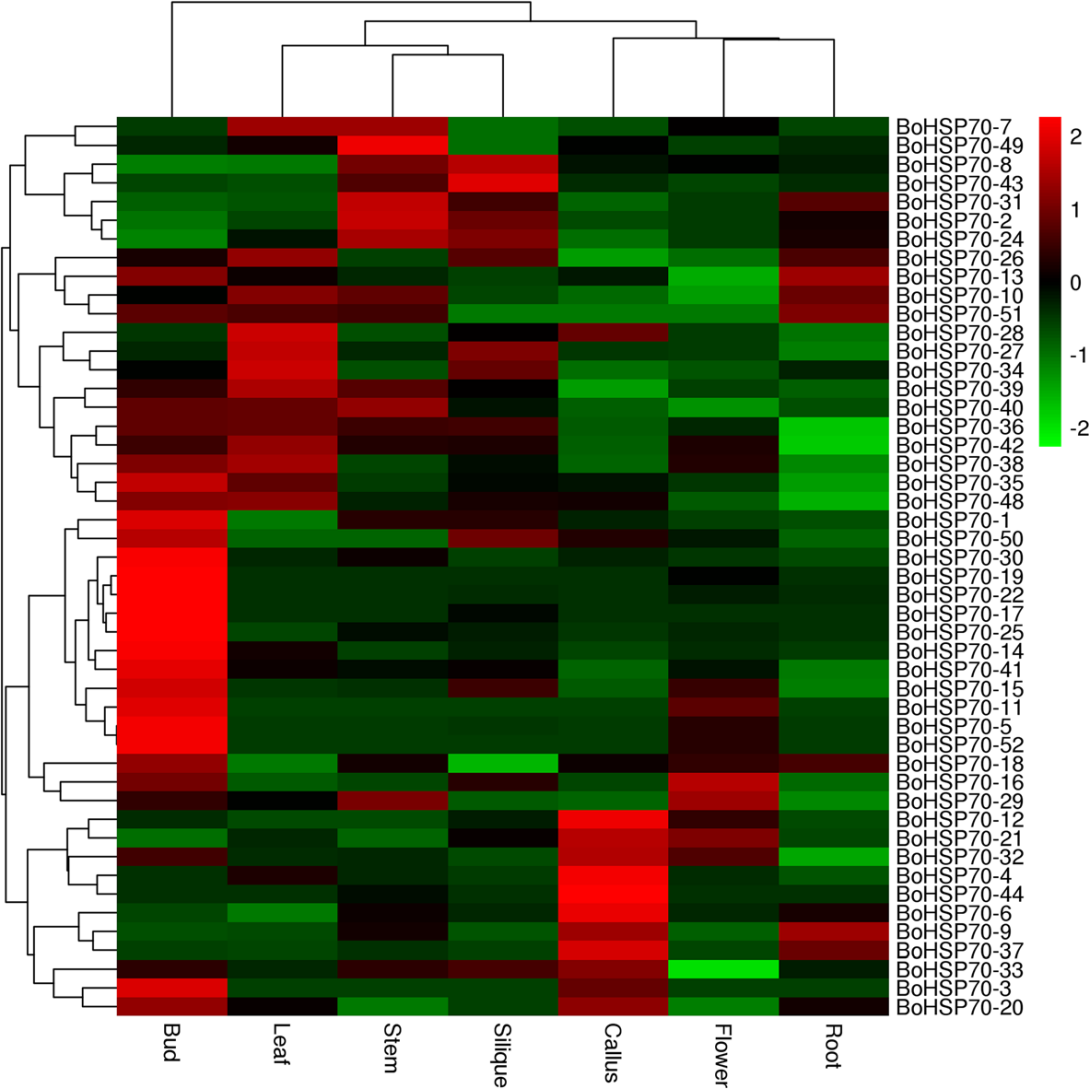
Heat map representation and hierarchical clustering of *BoHSP70* genes expression levels across roots, callus, siliques, stems, leaves, buds, and flowers

To obtain expression profiling of the *BoHSP70* genes, RNA-seq data from seven tissues (roots, stems, leaves, buds, flowers, calluses and siliques) were used in the expression analysis. As a result, a higher expression level of the *BoHSP70* genes was observed in bud tissue than the other tissues (Fig. 5). Those 52 identified *BoHSP70* genes were actively expressed in at least one of the six tissues (Fig. 5). High expression levels of *BoHSP70–30*, *BoHSP70–19*, *BoHSP70–22*, *BoHSP70–17*, *BoHSP70–25*, *BoHSP70–52*, and *BoHSP70–5* were observed in the buds. *BoHSP70–12*, *BoHSP70–4*, *BoHSP70–44*, *BoHSP70–46*, and *BoHSP70–37* were observed in calluses. *BoHSP70–28 and BoHSP70–34* were observed in the leaves. *BoHSP70–49* was observed in the stem, indicating its putative functions in the development and other physiological processes in buds. Nine *BoHSP70* genes were only upregulated in the buds (*BoHSP70–14*, *BoHSP70–30*, *BoHSP70–19*, *BoHSP70–22*, *BoHSP70–17*, *BoHSP70–25*, *BoHSP70–5*, *BoHSP70–41*, and *BoHSP70–52*), and four in calluses (*BoHSP70–12*, *BoHSP70–4*, *BoHSP70–6*, and *BoHSP70–44*). The genes that are highly expressed in plant tissues or organs are often found to be able to regulate target genes involved in the processes of plant growth and development.

**Fig. 6:**
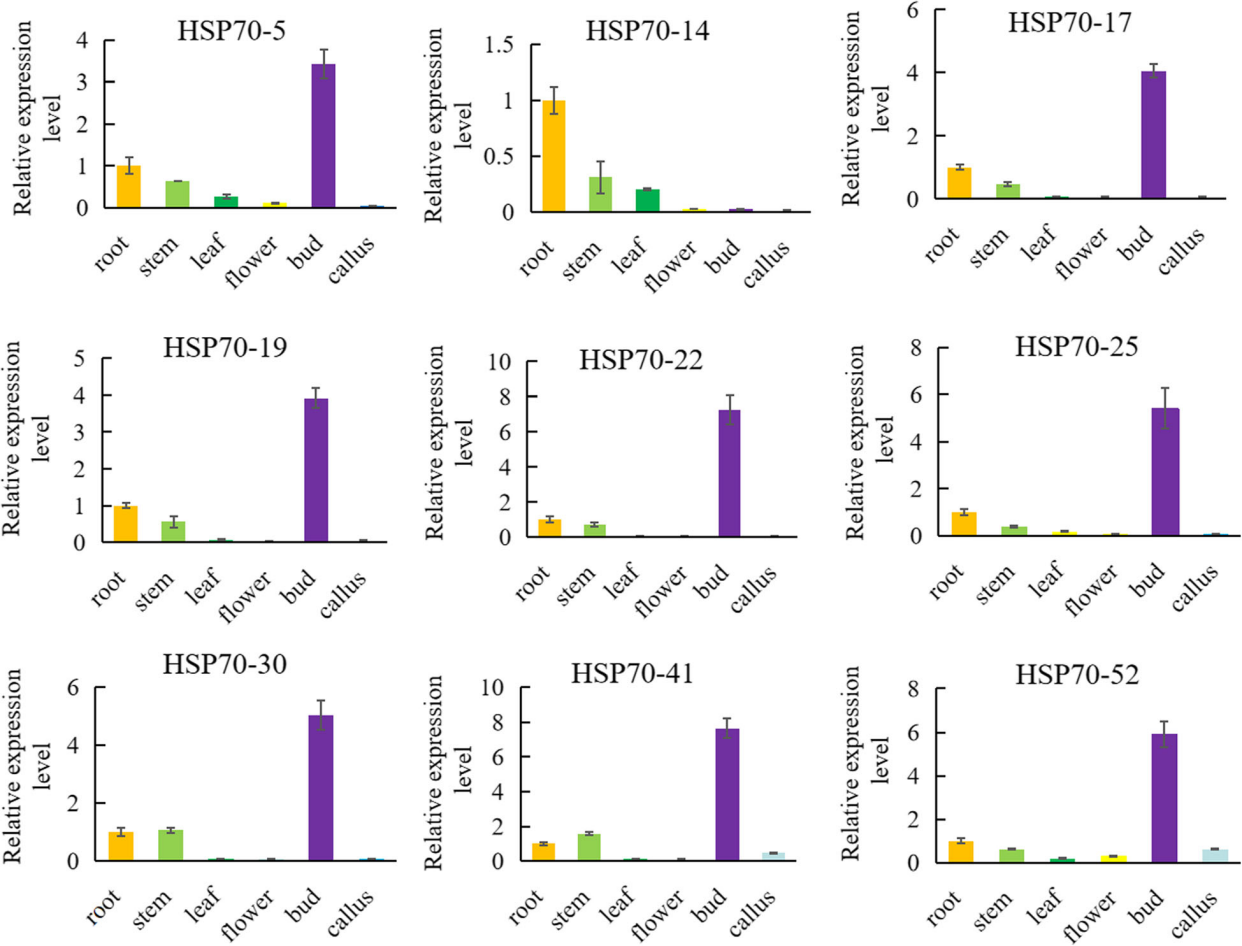
Expression analysis of the *BoHSP70* genes in different tissues. qRT-PCR analysis of the *BoHSP70* transcript levels in the roots, stems, leaves, flowers, buds and calluses. Data are presented as the means ± SD

In this study, we identified development-related *BoHSP70* genes at the transcription level in the cabbage genome, and nine genes (*BoHSP70–5*, *14*, *17*, *19*, *22*, *25*, *30*, *41*, and *52*) were selected in buds that were upregulated, downregulated or not different in other tissues based on the normalized FPKM values. qRT-PCR was conducted to verify the gene expression patterns of the *BoHSP70* in six different tissues, including bud, leaf, stem, callus, flower and root. *BoHSP70–5*, *14*, *17*, *19*, *22*, *25*, *30*, *41*, and *52* were primarily expressed in the buds. These transcripts could hardly be detected in leaves, flowers, and calluses, which was consistent with the results from RNA-seq analysis. Thus, we further confirmed their preferential expression as shown in Fig. 6. From this result we can find that eight genes detected by qRT-PCR are roughly consistent with the RNA-seq analysis, which further confirmed their preferential expression.

### Expression patterns of the *BoHSP70* genes in different periods of fertile and sterile buds and qRT-PCR verification

**Fig. 7:**
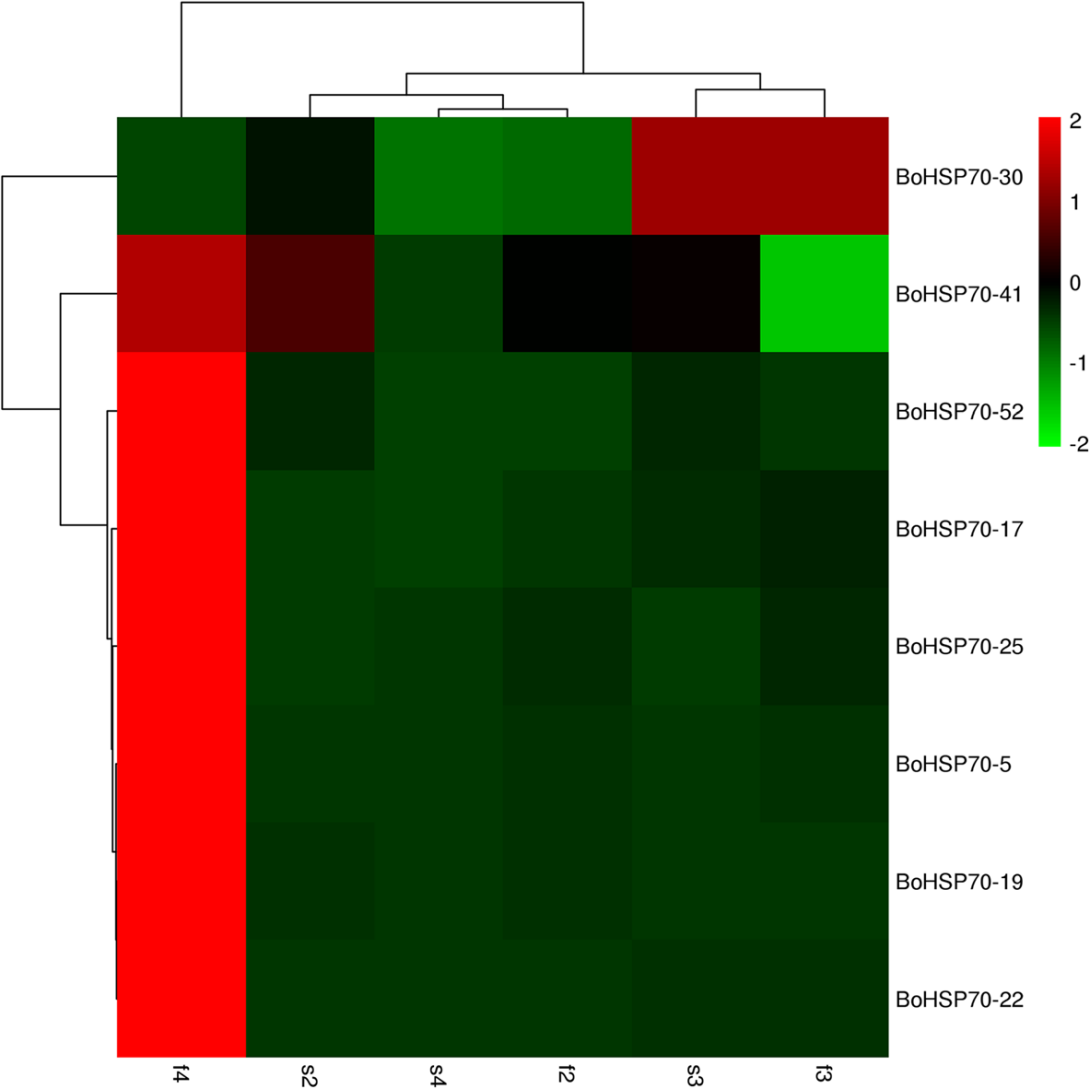
Heat map representation and hierarchical clustering of *BoHSP70* genes expression levels in different periods of cabbage bud. f2: tetrad stage of the fertile buds, f3: microspore period of the fertile buds, f4: binuclear phase of the fertile buds, s2: tetrad stage of the sterile buds, s3: microspore period of the sterile buds, s4: binuclear phase of the sterile buds

We found that some *BoHSP70* genes were specifically expressed in buds from the heat map in different tissues. To verify the specific role of these genes in flower development, RNA-seq data from six samples divided into three stages based on the developmental stages of the male gamete in which the female gamete is normal (f2: tetrad stage of the fertile buds, f3: microspore period, f4: binuclear phase; s2: tetrad stage of the sterile buds, s3: microspore period sterile, and s4: binuclear phase sterile) were used in the expression analysis. As shown in Fig. 7, *BoHSP70–5*, *17*, *19*, *22*, *25* and *52* were highly expressed at the binuclear phase (f4), while the low level of expression at the binuclear phase was significant (s4). *BoHSP70–41* had a significant low level of expression at the microspore period (f3).

**Fig. 8:**
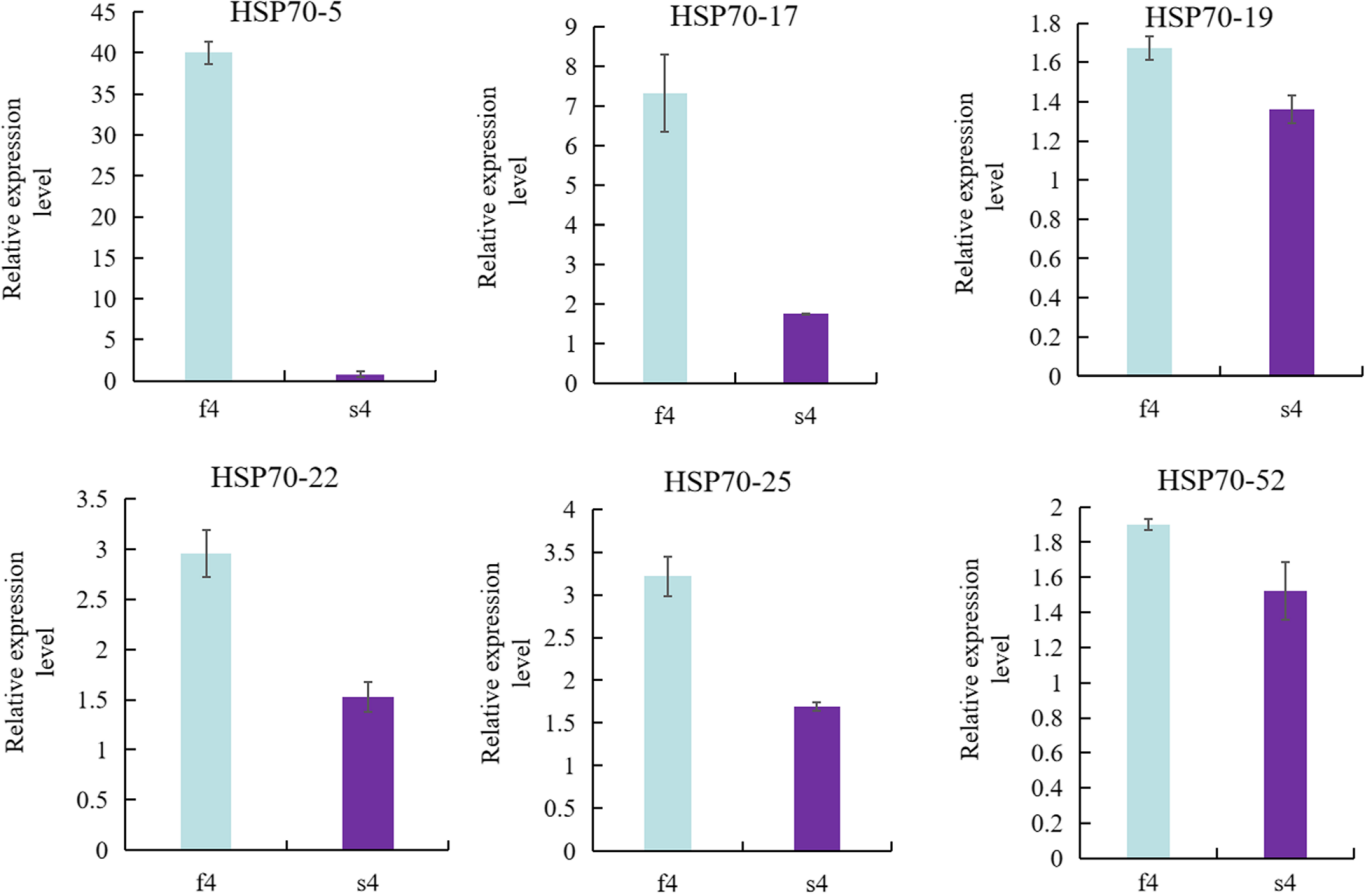
Expression analysis of the *BoHSP70* genes in the different periods of buds. Data are presented as the means ± SD. f4: binuclear phase of the fertile buds, s4: binuclear phase of the sterile buds

In this study, we identified developmental-related *BoHSP70* genes at the transcriptome level in the cabbage genome, and six genes (*BoHSP70–5*, *17*, *19*, *22*, *25*, and *52*) were selected in buds that were upregulated at the fertile binuclear phase, while they were downregulated at the sterile binuclear phase based on the normalized FPKM values. qRT-PCR was conducted to verify the gene expression patterns of *BoHSP70* at the binuclear phase. As shown in Fig. 8, in the binuclear phase, all six genes were highly expressed in the fertile buds compared to the sterile buds. These results were consistent with the data of the RNA-seq analysis, and the high expression of *BoHSP70* in f4 is likely to be highly expressed in stamens. Thus, those genes may be involved in stamen development.

### Subcellular localization of *BoHSP70–5*

**Fig. 9:**
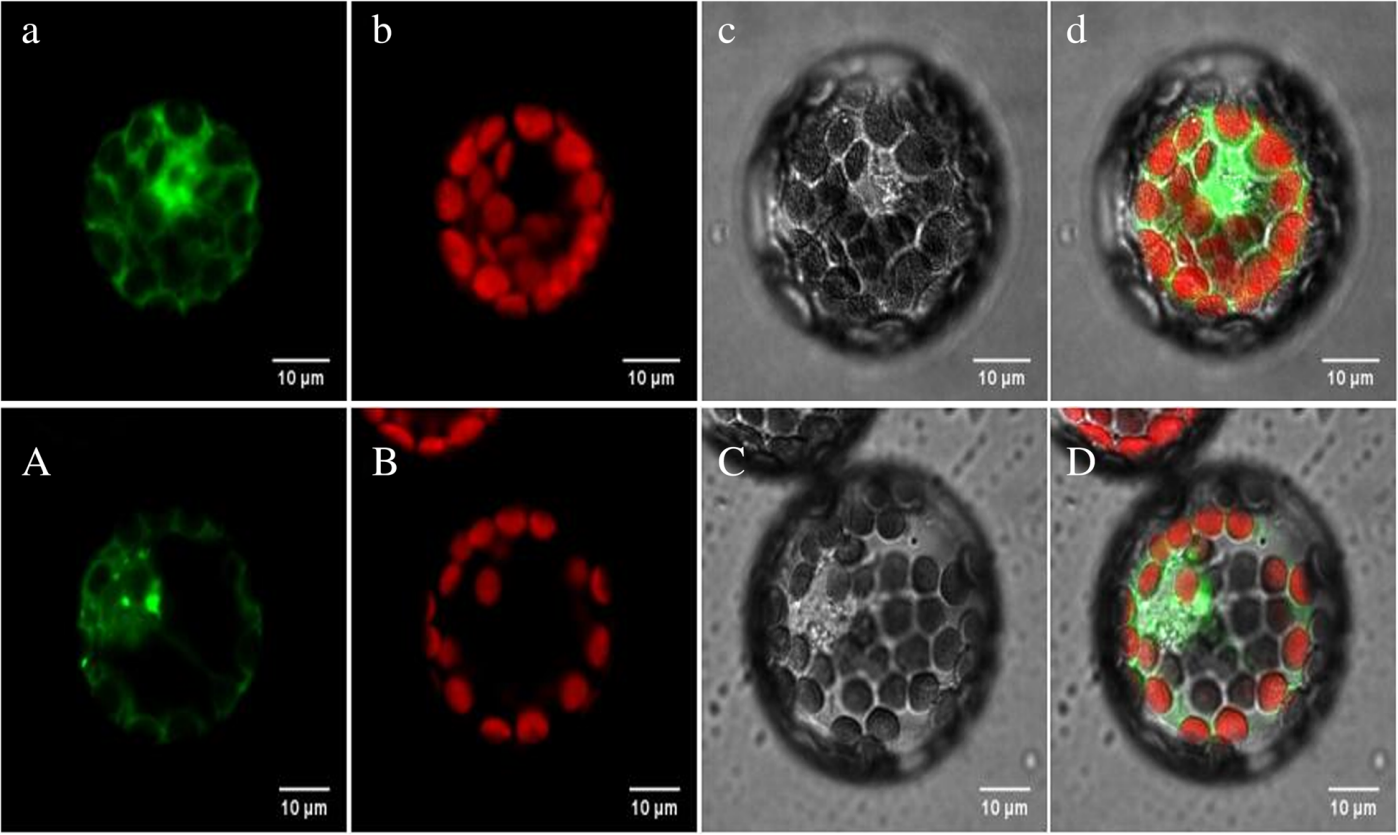
Subcellular localization of *BoHSP70–5* in Arabidopsis protoplast. a, b, c, d: Control GFP channel, Chloroplast channel, Bright, and Merge. A, B, C, D: Target protein GFP channel, Chloroplast channel, Bright, and Merge

Based on the results of the expression in different periods of buds (Fig. 7 and Fig. 8.), the *BoHSP70–5* gene expression in fertile was 40 times higher than that in sterile buds, the difference was significant between fertile and sterile buds. In silico subcellular localization prediction using Plant-mPLoc suggested that BoHSP70–5, 17, 19, 22, 25 and 52 proteins were localized in the cytoplasm. To further characterize the subcellular localization of the *BoHSP70–5* gene, was introduced into the pBWA(V)HS-GFP translational fusion construct. The recombinant pBWA(V)HS-*HSP70–5*-GFP fusion was infiltrated into the protoplast cells of Arabidopsis. pBWA(V)HS-GFP was used as a positive protein control and was detected in the nucleus and cytoplasm (Fig. 9 d). The GFP signal of *BoHSP70–5* was observed exclusively in the cytoplasm, suggesting that this *BoHSP70–5* was a cytoplasmic protein (Fig. 9 D), consistent with the in silico prediction results.

## Discussion

A previous analysis of the *HSP70* gene family was performed in the model plant *Arabidopsis thaliana* [23, 24]. However, this gene family has not been systematically characterized in cabbage. Therefore, we performed a whole-genome analysis of the *BoHSP70* gene family in cabbage, including an analysis of their phylogeny, chromosomal location, gene structure, conserved motifs and expression profiles. Based on the phylogenetic, gene structure, and motif analysis of the *BoHSP70* genes in cabbage, we discovered that the most closely related members in the same categories share similar exon/intron structures and intron numbers, which was similar to the *HSP70* family genes in soybean [13]. For example, in the class I, *BoHSP70* members contain two exons, while those in the class VII subfamily contain more than four exons. In the terminal branch of the phylogenetic tree, the number of exons/introns was similar in one of the sister pairs. However, there was still one sister pair that showed changes in their intron/exon structure and numbers, for example, *BoHSP70–2* and *BoHSP70–3*. These findings indicated that some intron loss, along with intron gain events, might have occurred during the structural evolution between the two families of the *BoHSP70*-encoding genes. The same situation was also revealed using motif analysis, and the type and number of motifs were similar in proteins within the same subfamily but differed from the proteins in the other subfamilies.

We also investigated gene duplication events to further understand the expansion mechanism of the *BoHSP70* family genes. The duplications of individual genes, chromosomal segments, or entire genomes have been major forces in the evolution of plant genome structure and content during the process of genome evolution [25–28]. A tandem duplication event is confirmed by the presence of two or more neighboring genes on the same chromosome, while a segmental duplication event is defined as a gene duplication on different chromosomes [29]. This study indicates that the *BoHSP70* family genes possess a higher whole genome duplication ratio (46%, 24 of 52 genes) and a lower tandem duplication ratio (4%, 2 of 52 genes) in cabbage. This is consistent with a previous study showing that tandem duplications have been rare in the expansion of the GRF, HSP and HSF families [30, 31]. To verify whether Darwinian positive selection was involved in the *HSP70* gene divergence after duplication, the nonsynonymous (Ka) versus synonymous (Ks) substitution rate ratios were calculated for the 26 paralogous pairs [32]. A Ka/Ks ratio significantly lower than 0.5 suggests a purifying selection for both duplicates. The low Ka/Ks ratio indicates that all the gene pairs, with the exception of two (*BoHSP70–36*/*BoHSP70–48* and *BoHSP70–36*/*BoHSP70–42*), might have evolved under the influence of purifying selection.

An analysis of gene expression patterns can be used to some extent to predict the molecular functions of genes involved in different processes [33]. A previous study established that HSP70s are expressed in different tissues and organs and are also expressed when treated with salt, drought and heat [34–38]. Our heatmap data showed that most *BoHSP70* genes were expressed in different cabbage tissues. These results indicate that they may participate in growth and development. It was determined that most of the paralogous pairs with high sequence similarity had similar expression patterns in different tissues. For instance, the *BoHSP70–5* and *BoHSP70–52* pair, the *BoHSP70–5* and *BoHSP70–19*, *BoHSP70–5* and *BoHSP70–11*, and *BoHSP70–35* and *BoHSP70–41* pairs showed strong expression in the bud. A divergence in expression was also found in the paralogous pairs. For example, in the *BoHSP70–13* and *BoHSP70–40* pair, *BoHSP70–13* was highly expressed in the roots, flowers and buds, with little or no expression in the other tissue types. However, its paralog, *BoHSP70–40*, was not only expressed in the roots, flowers and buds but also had obvious expression in the stems. It is interesting to note that nine genes were highly upregulated in buds and downregulated in the other five tissues, which indicates that those genes might be primarily involved in bud growth. However, the gene expressions profiles, which in the different tissues usually tended to be different, some bud-regulated genes were downregulated in the flowers and roots, which indicated that two sets of *BoHSP70* genes were involved in tissue development, respectively.

For the *BoHSP70* gene members, we were particularly interested in those that might play crucial roles in bud development. Sheoran et al. and Frank et al. indicated that both low and high molecular weight HSPs have been found to be expressed in the early and late stages of pollen development in various plant species [39, 40]. One hypothesis is that the activation of *HSP* gene expression during plant development is correlated to developmental programs rather than to the response of the plant under stressful environmental conditions [41, 42]. During pollen formation, HSPs may function as molecular chaperones for the folding/refolding of proteins involved in meiosis and tetrad formation [43]. In this study, RNA-seq data from six samples divided into three stages based on the developmental stages of the male gamete in which the female gamete is normal were used in the expression analysis. Those six *BoHSP70* genes in different periods of buds revealed that *BoHSP70–5*, *17*, *19*, *22*, *25*, and *52* were highly expressed at the binuclear phase in fertile buds (f4), low or no expression in other bud periods (f2, s2, f3, s3, s4) suggesting that those genes may be involved in pollen development, especially binuclear pollen. According to evolutionary classification, these six genes are family-specific genes in cabbage, which are far from homologous with *Arabidopsis thaliana*, therefore, subcellular localization was performed by selecting *BoHSP70–*5 of 40-fold different expression between fertile and sterile buds, the result showed that BoHSP70–5 was a cytoplasmic protein. Further experimental validations would broaden the understanding of the *BoHSP70* functions in stamen development at binuclear pollen.

## Conclusions

The cabbage (*Brassica oleracea* L.) genome contains 52 members of the *BoHSP70* family genes. Our research in this study identified and characterized the *BoHSP70* family genes and evaluated their gene and motif structures, their evolutionary histories, and their expression patterns in various cabbage tissues and different period of buds. Our findings will be helpful for further studies on the functions of these important transcription factors in various growth and developmental processes in cabbage. The overall description of this family genes and its potential involvement in pollen growth and development will facilitate further research on the *HSP70* family genes, particularly in regards to their evolutionary history and biological functions.

## Methods

### Genome-wide identification of the BoHSP70 genes

The whole-genome cabbage protein sequences were downloaded from the *Brassica oleracea* Genomics Database (www.ocri-genomics.org/bolbase/blast/blast.html). The Hidden Markov Model was download from the Pfam (http://www.sanger.ac.uk/Software/Pfam/) database (Pfam:PF00012) and used, and the protein data were scanned using hmmer 3.0 [44]. The first part of the candidate genes was obtained by preliminary screening according to an e-value < 0.01. To obtain the second part of the candidate gene, the HSP70 protein sequences of Arabidopsis were downloaded from the NCBI (https://www.ncbi.nlm.nih.gov/) as a query and submitted as a query in a BLASTP (*P* = 0.001) search. The results were screened with the criteria of an e-value <10e^− 10^ and match length > 100. The two candidate gene sets were combined; the protein sequence of the candidate gene was scanned using NCBI-CDD search (https://www.ncbi.nlm.nih.gov/cdd), and the family members are further identified based on the domain. The subcellular locations were predicted using Cell-PLoc 2.0 (http://www.csbio.sjtu.edu.cn/bioinf/Cell-PLoc-2/) [45].

### Conserved motif and gene structure analysis of the BoHSP70 genes

The amino acid sequences were subjected to “predict the domain and motif analyses” online using MEME (http://meme.sdsc.edu/meme/website/intro.html) [46], with the following parameters: number of repetitions: any; maximum number of motifs: 15; and the optimum motif widths: 6–200 amino acid residues. The nucleotide sequence and genomic sequence of the *HSP70* gene were stored in FASTA format, and the online tool Gene Structure Display Server (GSDS) was used to draw the gene exon-intron structure (http://gsds.cbi.pku.edu.cn/) [47].

### Construction of the phylogenetic tree

Based on the protein sequence, we used ClustalW to simultaneously align using MEGA5.0 to construct a phylogenetic tree based on the results of the joint NJ (Neighbor-join) (bootstrap = 1000), combined with cabbage, Arabidopsis, soybean and rice data (Additional file 1: Table S1) for phylogenetic tree analysis [48], the protein sequences of Arabidopsis, soybean, and rice HSP70 were acquired from Phytozome (Joint Genome Institute, JGI) (https://phytozome.jgi.doe.gov/pz/portal.html).

### Localization analysis of the BoHSP70 genes

The chromosomal distribution image of the *BoHSP70* genes was generated using Map Chart software based on the chromosomal position information provided in the genomic annotation file. Some of the genes located in the scaffold failed to be mapped to chromosomes, and random genes were not shown in the image [49].

### Gene doubling duplication and Ka/Ks analysis

Gene doubling and tandem (tandem repeat) were performed using MCSCANX, and selected doubling events related to family genes were performed using Circos mapping, while providing the number of doubled genes (collinearity analysis - gene doubling analysis) and tandem genes (gene doubling analysis - tandem repeat analysis) [50]. The Ka/Ks analysis of the *HSP70* family genes was conducted using DNASPv5 [51].

### Expression analysis of the BoHSP70 genes using the RNA-seq data

To assay the *BoHSP70* gene expression profiles, Illumina RNA-seq data of various tissues, including roots, stems, leaves, bud, flowers, calluses and siliques were downloaded from the NCBI (GSM1052958–964). To normalize the gene expression values, the fragments per kb of exon per million mapped reads (FPKM) algorithm was used in this study. The gene expression levels were calculated using the FPKM value, and the default empirical abundance threshold of 1 FPKM was used to evaluate whether a gene was positively expressed or not. Finally, *BoHSP70* gene expression profiles were displayed in Additional file 2: Table S2 and Additional file 3: Table S3, and the heat maps of hierarchical clustering were constructed in the Omics Share (www.omicshare.com/tools/Home/Index/index.html).

### RNA isolation, cDNA synthesis and quantitative real-time PCR analysis

The total RNAs of different developmental stages of the flowers of the two cabbage cultivars were extracted using an RNA Prep Pure Plant Kit (Takara, Dalian, Liaoning Province, China), and reverse-transcribed using Superscript III Reverse Transcriptase (Takara) following the manufacturer’s instructions, and the cDNA was diluted to 50 ng/L with ddH_2_O for further examination. Real-time quantitative PCR (qRT-PCR) was conducted using SYBR Green Supermix (Takara) with a total volume of a 10 μL reaction system on a CFX Connect TM Real-Time PCR Detection System (Bio-Rad, Hercules, CA, USA). These nine predicted *BoHSP70*s were selected based on FPKM values obtained from expression analysis of *BoHSP70s* in the RNA-seq data, and the primer sequences are shown in Additional file 4: Table S4. All the qRT-PCR reactions were performed in three independent biological repetitions with a template-free control to check any contaminations, actin was used as the internal reference gene in cabbage. The relative expression levels were calculated using the 2^-△△Ct^ method and plotted [52].

### Subcellular localization

The reaction mixture (50 μL) of the gene amplification consisted of 5 uL buffer, 2 uLMg^2+^, 2 uL dNTP, 0.5 uL forward and reverse primer, 1 uL cDNA template, 2 uL P^+^, 2 uL P^−^and 2 U of Taq DNA polymerase (TaKaRa, Dalian, China), add ddH_2_O to 50 μL. The amplification was performed at 94 °C for 5 min, followed by 30 cycles of 94 °C for 30 s, 60 °C for 30 s, and 72 °C for 30 s, with two final extension at 72 °C for 10 min and 30 min at 16 °C. The full coding sequences of *BoHSP70–5* were PCR-amplified with the primers F and R (Additional file 5: Table S5), the amplification products were digested with BsaI and Eco31I, then inserted into a pBWA(V)HS-GFP vector, resulting in an N-terminal fusion with GFP under the control of the constitutive CaMV35S promoter. The fusion constructs were introduced into *Arabidopsis thaliana* protoplasts as previously described [53]. The fluorescence signals were detected using confocal laser-scanning microscopy C1 (Nikon, Tokyo, Japan).

## Additional files

Additional file 1: **Table S1.** The IDs of *Hsp70* genes from different plants. (XLS 338 kb)

Additional file 2: **Table S2.** FPKM values of 52 *BoHsp70* genes in various cabbage tissues. (XLS 29 kb)

Additional file 3: **Table S3.** FPKM values of 8 *BoHsp70* genes in different periods of buds. (XLSX 10 kb)

Additional file 4: **Table S4.** The primer sequences of 9 *BoHsp70* genes used for qRT-PCR. (XLS 22 kb)

Additional file 5: **Table S5.** The primer used for cloning of *BoHsp70–5*. (XLS 19 kb)

## Abbreviations

BLASTP: Basic local alignment search tool-protein
FPKM: Fragments per kilobase of transcript per million mapped reads
HSFs: Heat shock transcription factors
HSPs: Heat shock proteins
qRT-PCR: Quantitative real-time polymerase chain reaction
sHSP: Small heat shock proteins
